# A Sputtered Gig-Lox TiO_2_ Sponge Integrated with CsPbI_3_:EuI_2_ for Semitransparent Perovskite Solar Cells

**DOI:** 10.1021/acs.jpcc.5c03520

**Published:** 2025-08-31

**Authors:** C. Spampinato, G. Calogero, G. Mannino, S. Valastro, E. Smecca, V. Arena, P. La Magna, C. Bongiorno, E. Fazio, A. Alberti

**Affiliations:** † 312425 National Research Council-Institute for Microelectronics and Microsystems (CNR-IMM), Zona Industriale—Strada VIII no. 5, Catania 95121, Italy; ‡ Dipartimento di Scienze Matematiche ed Informatiche, Scienze Fisiche e Scienze della Terra (MIFT), Università Degli Studi di Messina, Messina 98166, Italy

## Abstract

In this study, we propose a novel approach where in an innovative porous grazing incidence atomic flux coupled with local oxidation (gig-lox) TiO_2_ electron transport layer (ETL), deposited by sputtering, is integrated with a fully inorganic CsPbI_3_:EuI_2_ perovskite. This combination is used as a photoactive layer in semitransparent perovskite solar cells (ST-PSC). The solvent-free, scalable porous oxide sponge-like in structure, is produced via a grazing-incidence Ti flux that undergoes localized progressive oxidation. The sponge offers ≈50% volume porosity that is available for perovskite infiltration. The result of the oxide-perovskite integration is a double-layer struc made of a perovskite-filled sponge covered by a cap of pure perovskite. The material has unique optical properties and structure as attested by spectroscopic ellipsometry and X-ray diffraction analyses. The double-layer structure exhibits semitransparency, and its reduced photoluminescence (PL) intensity relative to that of a single perovskite layer indicates efficient charge carrier injection into the porous TiO_2_. In perspective, the layer can be used in PSC as demonstrated by simulations.

## Graphical abstract

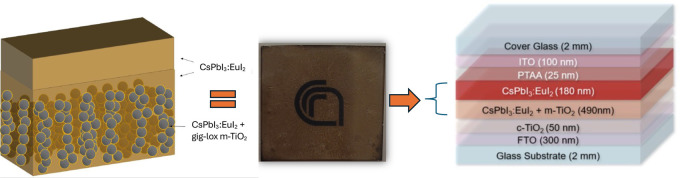

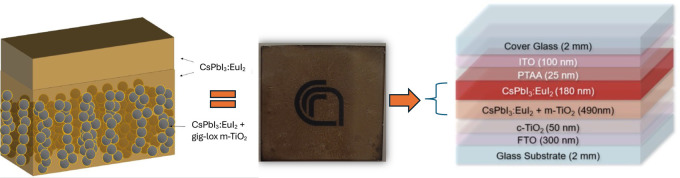

## Introduction

Perovskite materials have shown remarkable progress in recent years, particularly in photovoltaics and optoelectronics, due to their exceptional light-harvesting capabilities and tunable optoelectronic properties.^1^ Within this class, inorganic CsPbI_3_ has garnered significant attention for ST-PSC. This is attributed to its relatively large bandgap, which favors transmission of light in the visible range, and its thermal compositional stability compared to hybrid organic–inorganic perovskites.^2,3^ Furthermore, bandgap engineering through compositional modifications enables fine-tuning of the absorption edge, optimizing the trade-off between transparency and power conversion efficiency (PCE).^4^

Despite the intrinsic benefits of CsPbI_3_, including preparation by solution processing (e.g., spin coating and inkjet printing) and relatively high-power conversion efficiencies exceeding 21%,^5^ several challenges impede its large-scale use. These include its photoactive polymorphism instability under ambient conditions,^6^ hysteresis effects,^4^ and the complexities associated with upscaling.^7,8^ Strategies to mitigate these drawbacks include encapsulation protocols and additional processes for crystallographic stabilization.^9–11^

Among various stabilizing approaches, partial substitution of PbI_2_ with EuI_2_ has been shown to enhance both operational stability and device efficiency.^12^ However, incorporating Eu limits the practical thickness of the perovskite films, typically resulting in layers of only ∼200 nm.^12^ The benefits of adding EuI_2_ are reported in ref 13. Although thinner layers benefit from high transparency, their reduced thickness is insufficient to maximize photovoltaic performance in PSC.^14^ In contrast, standard CsPbI_3_ perovskites can readily form thicker layers, typically 500 nm or more, that yield good photovoltaic performance;^15^ however, these thicker films significantly compromise transparency, hindering their suitability for ST-PSC applications.^16,17^ In this work, we address these limitations by integrating a fully inorganic CsPbI_3_:EuI_2_ photoactive layer with a nanoporous TiO_2_ ETL fabricated via a gig-lox process.^18^ This solvent-free, upscalable gig-lox methodology allows for the deposition of thick and transparent TiO_2_ scaffolds, thereby improving mechanical stability and resilience against environmental species action, while preserving the semitransparent character of the perovskite-based device. Specifically, the innovative application of gig-lox TiO_2_ enables high semitransparency and efficiency, demonstrated by simulations, thanks to its porosity (≈50%) as measured by ellipsometry according to the model in ref 19.

## Experimental Methods

### TiO_2_ Deposition

Spongy gig-lox TiO_2_ is grown using a technique in which Ti species generated in an Ar-based plasma by sputtering from a metallic source are deposited onto a substrate in grazing incidence undergoing gradual local oxidation during the growth process. This technique was previously described in ref 18 with further angular optimization to enhance the layer’s porosity.^20^ This approach offers several advantages. First, a high deposition rate of 4 nm/min is achieved by establishing a metallic plasma at the source side, which prevents charging effects from surface oxidation. Second, it promotes the progressive local oxidation of the deposited atoms at the anode side. Lastly, the tilted (off-axis) Ti flux creates a shadowing from the initial seeds, resulting in additional meso-porosity along the deposited layer, in addition to the nanoporosity achieved by a high-pressure of Ar.^21^ Importantly, the overall porosity remains unchanged during postdeposition thermal treatments, and this has been demonstrated for layer thicknesses of up to 1000 nm as discussed in ref 22. In this paper, we employ a ≈450 nm thick TiO_2_ film, the typical thickness of chemically deposited mesoporous layers in PSC.^23^

### Perovskite Film Fabrication

For the perovskite solution, 1 M PbI_2_ and 1 M CsI, from Tokyo Chemical Industry were mixed. These compounds were dissolved in a mixed solvent made of DMF and DMSO, in a volumetric ratio of 3:1. Simultaneously, a solution of EuI_2_ from Sigma-Aldrich was prepared at a concentration of 0.1 M by using the same mixed solvent. Both solutions were subjected to stirring at room temperature for 1 h. For the CsPbI_3_:EuI_2_ samples, 1 mL of the PbI_2_/CsI solution was mixed with 0.5 mL of the EuI_2_ solution to achieve the desired stoichiometry. These resulting mixtures were additionally stirred for 1 h. Along the entire procedure, ambient air maintained a relative humidity of approximately 35%.

To deposit the perovskite films, spin-coating in a N_2_ environment was done over thermally pretreated (500 °C 30 min) gig-lox TiO_2_ substrates. The deposition process consists of two sequential steps: initially spinning at 1000 rpm for 10 s and a subsequent round at 2000 rpm (round per minute) for 25 s^24^

### Scanning Electron Microscope

A scanning electron microscope (Zeiss Gemini II FE-SEM) in plan-view configuration operating at an accelerating voltage of 30 kV and at a working distance of 4 mm in transmission mode and at an accelerating voltage of 3 kV for the SEM images was used to investigate the morphology of the gig-lox TiO_2_ and perovskite layers.

### Transmission Electron Microscopy Analysis

To prevent dissolution of the perovskite layer due to the use of water in standard preparation procedures, the gig-lox TiO_2_ layer was mechanically scratched from the glass substrate directly onto a Transmission electron microscopy (TEM) carbon grid. TEM images were obtained employing a probe-corrected Jeol ARM200 microscope, which was equipped with a JEOL 100 mm^2^ energy dispersive X-ray (EDX) detector. The imaging process was conducted in scanning mode, utilizing a high-angle annular dark field (HAADF) detector with a 50 mrad aperture. The acquisition of EDX spectra was done in a spectrum image configuration, encompassing the entire film thickness, ranging from the surface down to the lower interface.

### X-ray Diffraction Analysis

X-ray diffraction (XRD) patterns were obtained using a SmartLab (Rigaku) diffractometer equipped with a 9 kW rotating anode KαCu source, working at 45 kV and 100 mA. For acquisitions, a HyPix-3000 detector was used. The recording step size for the patterns was set at 0.01°, with an acquisition speed of 0.1° per minute.^25^

### Spectroscopic Ellipsometry Analysis

The optical constants were measured using a J.A. Woollam VASE ellipsometer equipped with a rotating compensator, which enhanced measurement accuracy and allowed for the detection of any nonpolarized light components. The sample was placed in a sealed chamber maintained under a slight overpressure of nitrogen (N_2_) to prevent degradation from exposure to ambient humidity. Spectroscopic ellipsometry was performed over the photon energy range of 0.5–6.5 eV to develop a comprehensive optical model. Since the sample was deposited on a glass substrate, its influence was carefully accounted for, including the potential contribution of back-side reflections. The thickness of the perovskite layer was initially extracted using the Cauchy model in the transparent spectral region and held constant during subsequent fitting. A Kramers–Kronig consistent model was then constructed using a set of multiple critical point parabolic band oscillators to accurately describe the optical response of the layer.^26–28^

### Photoluminescence Spectroscopy Analysis

Photoluminescence (PL) measurements were conducted using an Arkeo (Cicci Research s.r.l.) instrument. Samples were excited with a green laser (532 nm) at a 45° incident angle, producing a circular spot 1 mm in diameter. After a 1000 ms, the PL signal was collected via a spectrometer.

### Device Simulation

Photovoltaic devices were simulated using the 1D electro-optical simulator Setfos (v5.5) by Fluxim,^29^ which self-consistently combines the drift-diffusion formalism for charge transport with the transfer-matrix formalism for optical absorption/transmission. The Air Mass 1.5 ASTM G-173–03 (included in the SETFOS database) is adopted as the reference spectrum for 1 sun illumination. The wavelength-dependent complex refractive indices (*n*(λ) and *k*(λ)) of the gig-lox TiO_2_ layer infiltrated and capped with perovskite were extracted from spectroscopic ellipsometry . The *n*(λ) and *k*(λ) for PTAA were taken from ref.^30^, those for F-doped tin oxide (FTO) and compact TiO_2_ from ref 31 and those for indium tin oxide (ITO) from the Setfos database. All layers, except glass, are modeled as optically coherent. The drift-diffusion model in steady-state mode is solved by letting Setfos automatically select the appropriate solver (Newton or Gummel) and the corresponding settings for the residuum and damping factors during the computation. The bandgap of the perovskite capping layer and the gig-lox TiO_2_ infiltrated with perovskite were set to the values extracted by PL analysis (1.75 and 1.78 eV, respectively). The optical properties of gig-lox TiO_2_ require an optimized model with respect to those of the conventional TiO_2_, as reported in ref 31. The remaining electrical parameters for all layers were taken from ref 32.

## Results and Discussion

Film deposition on glass was performed by spinning the CsPbI_3_:EuI_2_ solution at 1000 rpm (rounds per minute) as the first step. The spin coating speed (ranging from 1000 to 5500 rpm) at the second step was used as a parameter, and it has been found that the layer thickness is independent of it even using 2000 rpm, as reported in Table S1 and Figure S1 of the Supporting Information file. The thickness measured on glass via spectroscopic ellipsometry is 180 nm. Spin coating was also performed on a 460 nm thick gig-lox TiO_2_. Figure 1 shows the resulting layer viewed by TEM analysis taken on a scratched portion. The main result is a uniformly infiltrated^33^ TiO_2_ sponge with CsPbI_3_:EuI_2_ distributed throughout the whole TiO_2_ oxide thickness as revealed through by the mass-contrast in the scanning TEM image.

**Figure 1:**
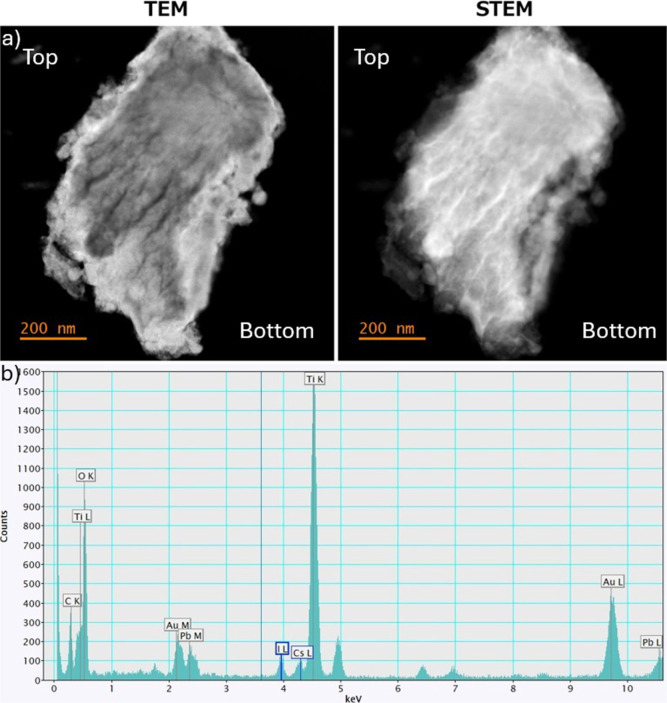
Transmission electron microscopy analysis: (a) sample portion in mass contrast. (b) EDX spectrum for element recognition. Sample grid produces the gold and carbon peaks.

In the image, the white areas arise from species with higher atomic mass, which indeed reflect the infiltration of the perovskite layer. Additionally, an EDX analysis (Figure 1b) was conducted to identify the most abundant chemical species. It shows the presence of titanium, oxygen, cesium, lead, and iodine sharing the same thickness, which confirms the in-depth intermixing of the two materials (gold and carbon peaks are typical of the supporting grid).

By the spin-coating process, we achieved the complete infiltration of a CsPbI_3_:EuI_2_ solution within the gig-lox TiO_2_. We deliberately promoted the formation of a perovskite cap layer on top, as described below. The presence of a cap was carefully controlled using a spin-coating process at 2000 rpm, as confirmed by SEM analyses in plan-view.

Figure 2a shows the CsPbI_3_:EuI_2_ surface morphology of the cap. Through some uncovered regions, the surface of the gig-lox TiO_2_ underneath is visible. As a reference, Figure 2b displays the morphology of a bare gig-lox TiO_2_ sample, with its typical grain size, markedly different from that of CsPbI_3_.^18^ Based on this evidence, the layer sequence is schematically depicted in Figure 2c. This two-layer structure consists of a gig-lox TiO_2_ infiltrated with CsPbI_3_:EuI_2_ and a cap layer. The composition of this two-layer was investigated by X-ray diffraction analyses. In Figure 3a, the diffractograms collected at various angles of incidence on glass (a) and on gig-lox TiO_2_ (b) are shown.

**Figure 2:**
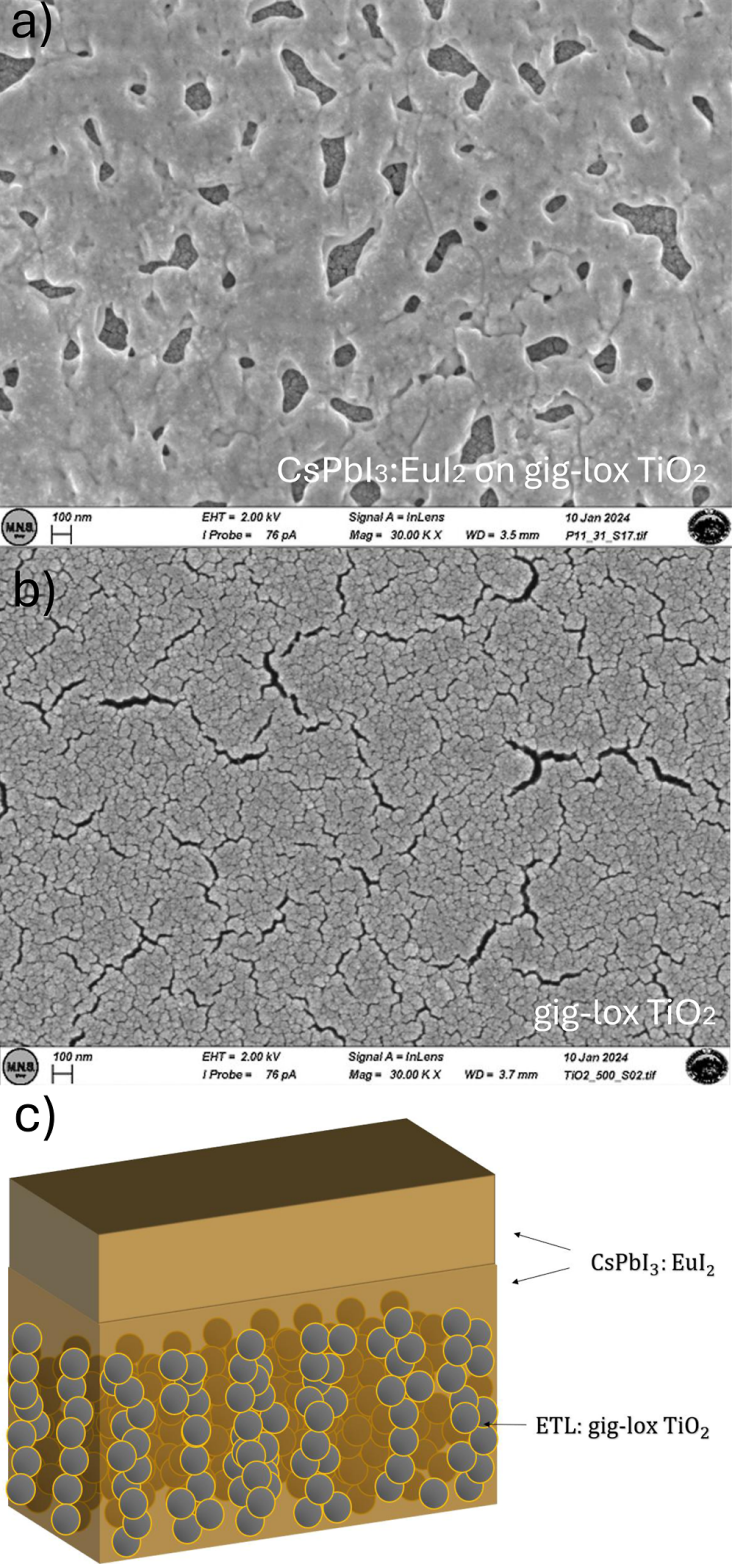
a) SEM plan-view of CsPbI_3_:EuI_2_ infiltrated into gig-lox TiO_2_. (b) SEM plan-view of the reference bare gig-lox TiO_2_. (c) Schematic of the gig-lox TiO_2_ structure with infiltrated CsPbI_3_:EuI_2_ and the CsPbI_3_:EuI_2_ cap. The sponge offers ≈50% volume porosity for perovskite infiltration, as measured by spectroscopic ellipsometry according to the model in ref 19.

**Figure 3:**
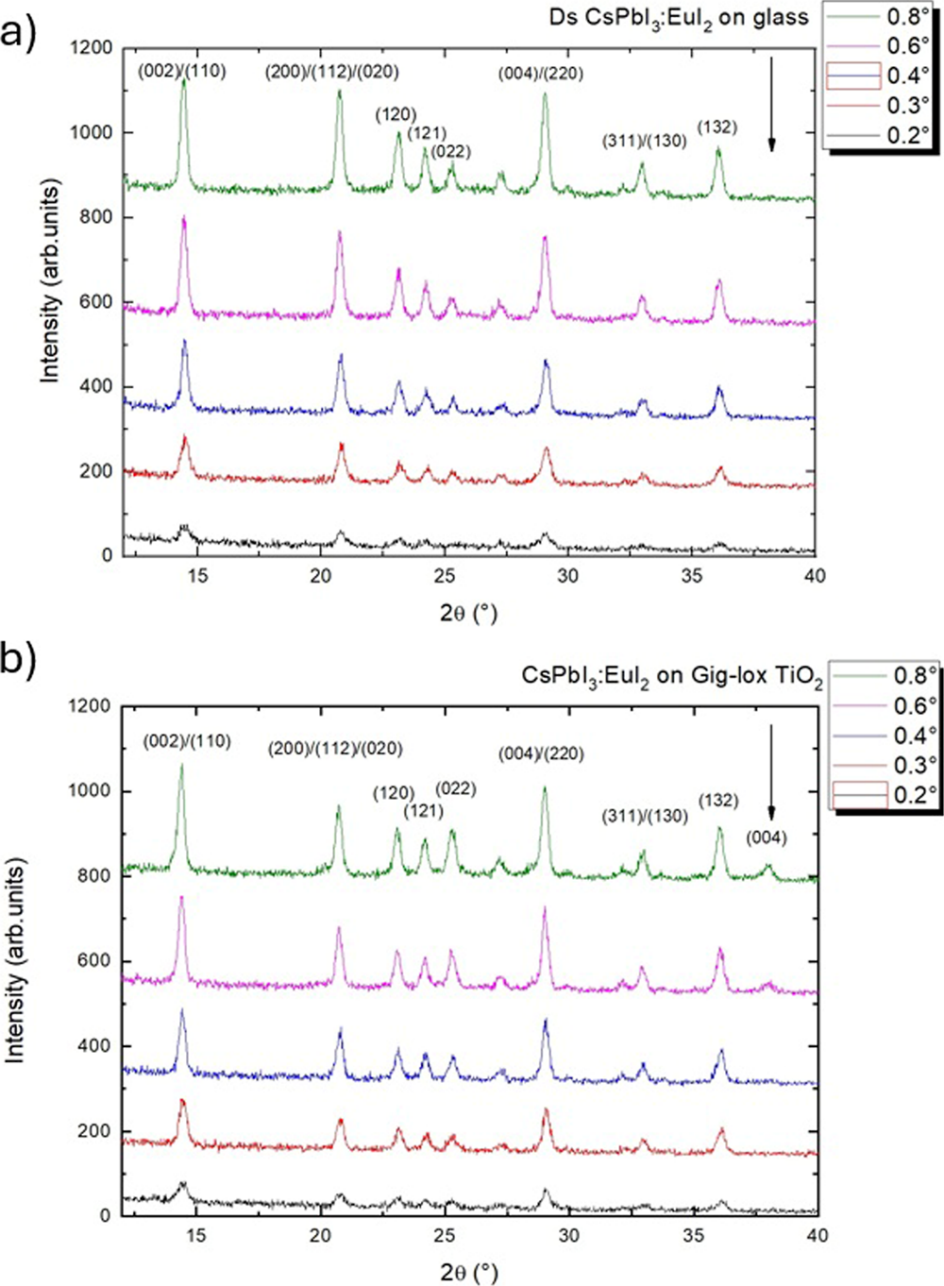
a) Diffraction patterns at different angles (0.2°- 0.3°- 0.4°- 0.6°- 0.8°) for CsPbI_3_:EuI_2_ layer deposited on glass. (b) CsPbI_3_:EuI_2_ layer deposited on gig-lox TiO_2_.

The structure preservation of CsPbI_3_:EuI_2_ once infiltrated into gig-lox TiO_2_ is demonstrated by the presence of the typical crystallographic planes compared to CsPbI_3_:EuI_2_ deposited on a glass substrate. In the grazing angle scan at 0.6° incident angle (Figure 3a), a distinct diffraction peak emerges at 2θ = 37.9°, which corresponds to the crystallographic (004) planes of anatase-TiO_2_. This peak is not detectable at lower grazing angles. The penetration depth calculated^34^ as the distance at which the X-ray beam intensity decreases to 1/*e* suggests that a capping layer of pure perovskite, ≈150 nm thick, is formed on top of the gig-lox TiO_2_/CsPbI_3_:EuI_2_. From the full width at half maximum (FWHM) of the diffraction peaks and, according to the Debye–Scherrer relation,^35^ we measured an average crystal size of 49.2 ± 9.2 nm in the CsPbI_3_:EuI_2_ reference on glass and 36.2 ± 9.1 nm in the CsPbI_3_:EuI_2_ on gig-lox TiO_2_. This shrinkage is attributed to the intercalation into the gig-lox TiO_2_ structure that is highly branched at the nanometer scale.^18^ Through Rietveld refinement (data are listed in Table 1 and 2.), the infiltrated perovskite structure is compared to the reference structure in the film on glass, as listed in Table 2. For the refinement, an orthorhombic 62:*Pnma* (χ^2^ = 1.3) crystal symmetry was considered.

**Table 1:** Peak Position and Full Width at Half Maximum (FWHM) of the main contributions in the reference CsPbI_3_:EuI_2_ on glass substrate and in the CsPbI_3_:EuI_2_ infiltrated into gig-lox TiO_2_

CsPbI_3_:EuI_2_ on glass	2θ [°]	FWHM [°]
(002)/(110)	14.37	0.18
(200)/(112)/(020)	20.67	0.16
(120)	23.09	0.18
(121)	24.16	0.22
(022)	25.23	0.20
(004)/(220)	28.98	0.20
(311)/(130)	32.90	0.17
(132)	36.04	0.23

CsPbI_3_:EuI_2_ on gig-lox TiO_2_	2θ [°]	FWHM [°]
(002)/(110)	14.37	0.19
(200)/(112)/(020)	20.64	0.19
(120)	23.12	0.19
(121)	24.52	0.25
(022)	25.28	0.22
(004)/(220)	28.38	0.37
(311)/(130)	32.98	0.22
(132)	36.07	0.26

**Table 2:** Lattice parameters of CsPbI_3_:EuI_2_ on glass substrate and CsPbI_3_:EuI_2_ infiltrated into gig-lox TiO_2_ from Rietveld refinement.

lattice parameters	CsPbI_3_:EuI_2_ on glass	CsPbI_3_:EuI_2_ on gig-lox TiO_2_
*a* [Å]	8.842	8.875
*b* [Å]	12.240	12.578
*c* [Å]	8.586	8.592
unit cell [Å^3^]	929	959

It is interesting to notice that the lattice parameters slightly increased in the perovskite infiltrated into the gig-lox TiO_2_ structure, resulting in an overall unit cell volume expansion of 3.2%. The results are consistent with the porous nature of the TiO_2_ substrate, with the fine pores allowing an inner adaptation of the intercalated perovskite, which is indeed free to expand with respect to a more constrained compact film. We investigated the two-layer structure also from the optical point of view by spectroscopic ellipsometry. The experimental data were acquired by placing the samples in a chamber filled with N_2_ to prevent degradation of the perovskite in humid air. The enclosure limited data collection to an angle of 70°. Figure 4 displays the experimental data and the model for two reference samples (i.e., (a) CsPbI_3_:EuI_2_ sample and (b) gig-lox TiO_2_ sample) and for two-layer structure: CsPbI_3_:EuI_2_ cap on the mixed CsPbI_3_:EuI_2_/gig-lox TiO_2_ sample (c). Remarkably, a satisfactory matching between the fitting model and experimental results was achieved across the whole investigated wavelength range.

**Figure 4:**
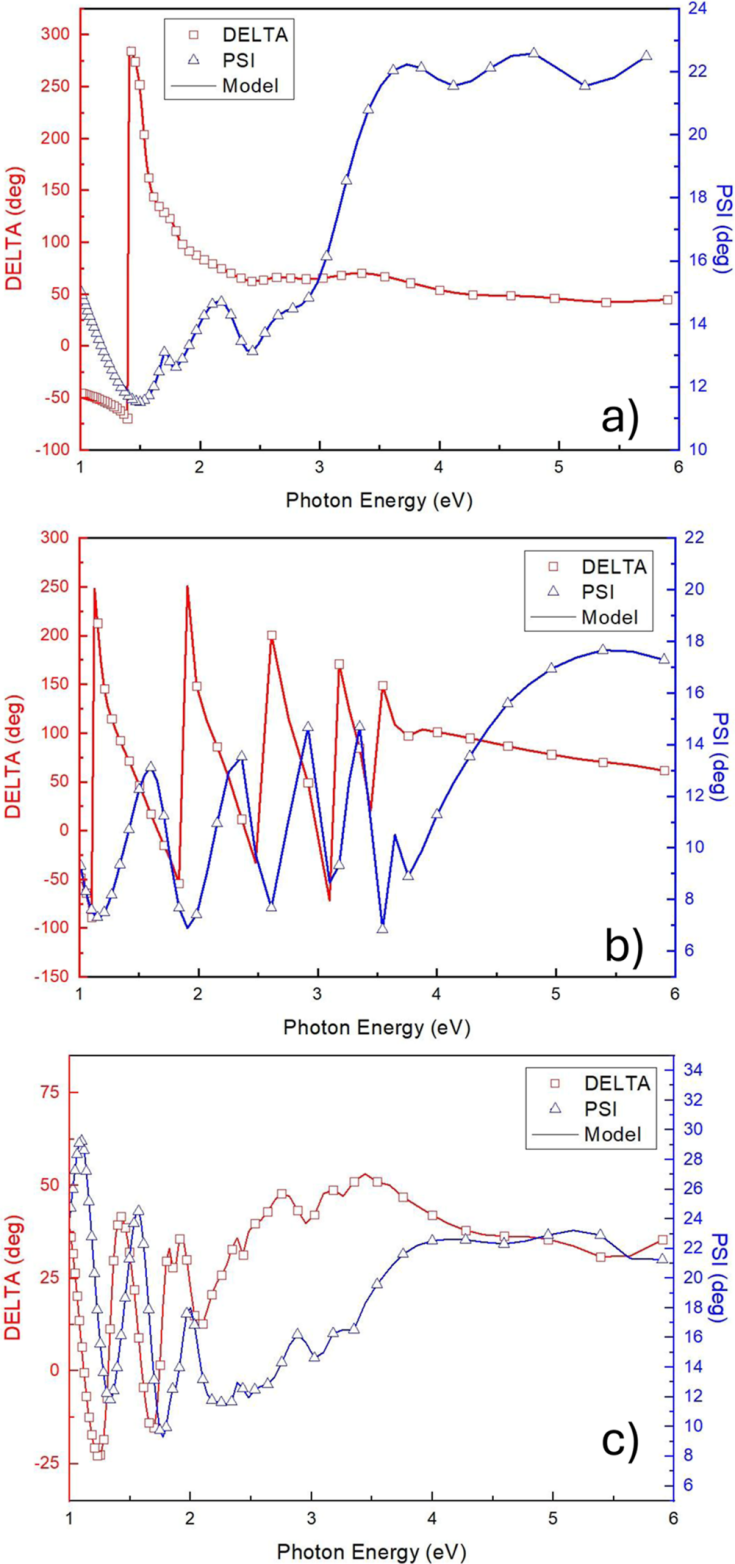
Spectroscopic ellipsometry: experimental (symbols) and model (lines) data at 70° angle of incidence for (a) CsPbI_3_:EuI_2_, (b) gig-lox TiO_2_, and (c) the two-layer structure: CsPbI_3_:EuI_2_ cap on mixed CsPbI_3_:EuI_2_/gig-lox TiO_2_.

It is noteworthy that the combined layer still retains features coming from the two layers of which it is composed. In particular, in the UV region (>4 eV) where TiO_2_ is transparent, the optical constants closely resemble those of the perovskite layer, whereas in the IR region (<1.5 eV) those of the gig-lox TiO_2_.

The absorption coefficient of the two-layer structure: CsPbI_3_:EuI_2_ cap on mixed CsPbI_3_:EuI_2_/gig-lox TiO_2_ (Figure 5a) was then calculated from the dielectric function using the equation in refs 36,37 .

**Figure 5:**
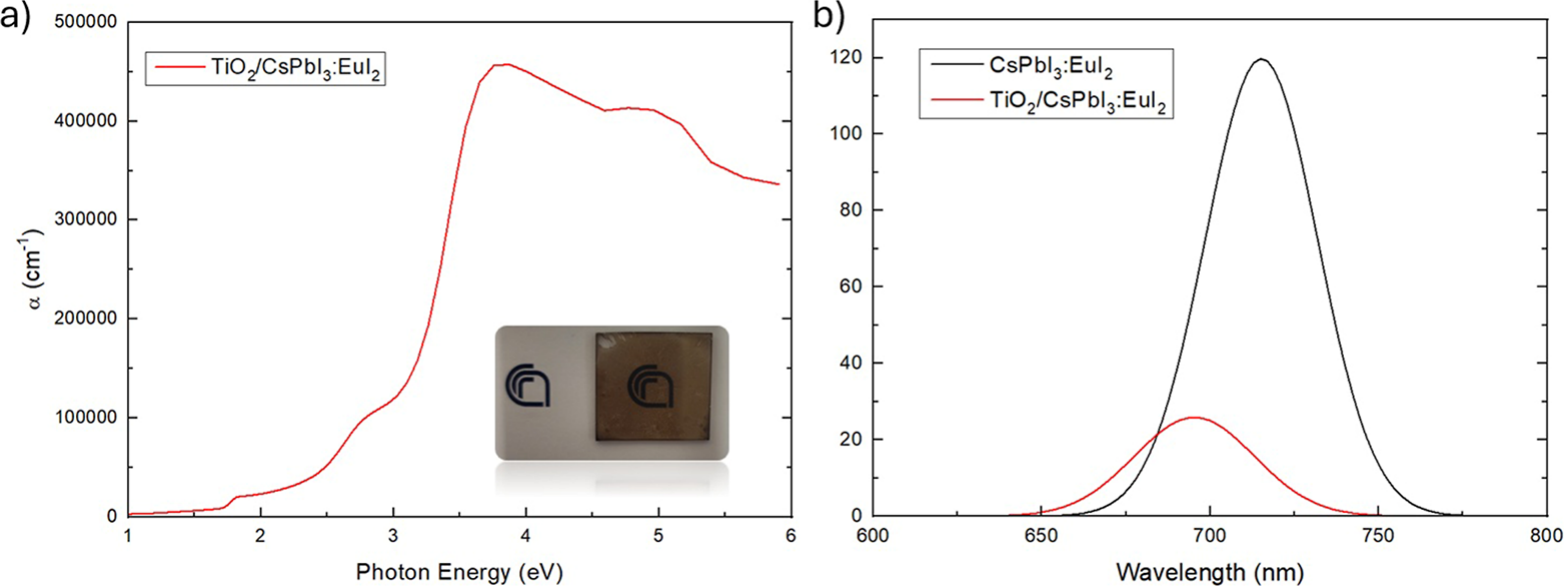
(a) Absorption coefficient of the entire two-layer structure: CsPbI_3_:EuI_2_ cap on mixed CsPbI_3_:EuI_2_/gig-lox TiO_2_. (b) PL spectra of reference CsPbI_3_:EuI_2_ on glass and on gig-lox TiO_2_.

A crucial aspect to consider is the region below the bandgap at 1.78 eV, where the material is expected to be transparent. In this region, despite slight absorption, the material maintains a low absorption coefficient, indicating its high optical quality. To investigate the applicability of the two-layer structure in PSC, it is crucial to probe the role that TiO_2_ plays in extracting charges. To achieve this, we measured the PL of the material in a N_2_ environment. Figure 5b shows the emission spectra of both the two-layer struc and the reference sample. The CsPbI_3_:EuI_2_ deposited on the gig-lox sample exhibits a significantly reduced PL intensity compared to that on glass, with a remarkable 84% decrease in emission. This substantial reduction in radiative recombination indicates that a high density of carriers is being extracted by the TiO_2_.

Moreover, in the reference on glass, the peak of PL is centered at 715 nm (1.75 eV), whereas the CsPbI_3_:EuI_2_ deposited on the gig-lox TiO_2_ has a peak at 695 nm (1.78 eV). This variation is attributed to the measured difference in the crystallite sizes, where smaller dimensions correlate with higher bandgaps^38–40^ and to the volume of the unit cell as discussed before. The findings support an efficient injection of charge carriers from the CsPbI_3_:EuI_2_ into the porous TiO_2_ thanks to branched interfaces,^36,41^ unlike in the reference layer CsPbI_3_:EuI_2_ on glass.

For applicative purposes, it might be of interest to evaluate how this two-layer structure can contribute to the development of PSC. We simulated a complete device using the Setfos simulation software^29^ (all computational details are reported in Experimental Methods). Currently, the technological realization of high-performance semitransparent perovskite-based devices remains highly challenging. Through advanced simulations, the aim is to provide a clear and quantifiable target for ongoing research efforts. This approach seeks to identify key parameters and design principles that can guide experimental development, ultimately facilitating progress toward achieving efficient, stable, and scalable semitransparent perovskite devices suitable for real-world applications.^42^ The 180 nm thick CsPbI_3_:EuI_2_ perovskite layer and the infiltrated CsPbI_3_:EuI_2_/gig-lox TiO_2_ layer are integrated into a simulated ST-PSC architecture similar to that reported in ref 32 as shown in Figure 6a. It consists of a planar n-i-p architecture on a glass substrate in which the perovskite layer is sandwiched between the 450 nm thick infiltrated CsPbI_3_:EuI_2_/gig-lox TiO_2_, acting as an ETL, and a 25 nm thick poly­[bis­(4-phenyl)­(2,4,6-trimethylphenyl)­amine (PTAA) film, acting as ETL. A 300 nm F-doped tin oxide (FTO) and a 100 nm indium tin oxide (ITO)^43^ are chosen as the transparent bottom and top electrodes, respectively. A glass layer is added on the ITO top electrode to consider the cover glass required for PSC encapsulation.^44^ The deposition of both ITO and PTAA follows the procedure reported in earlier studies.^32^ The thickness of the transport layers and electrodes was set based on values reported in the literature for similar ST-PSCs.^45^ The absorption spectra of the individual layers (Figure 6b) and the transmittance (Figure 6c) of the device are presented. Reflectance and transmittance data for the individual layers are reported in the supporting file Figure S2. Figure 6d displays the JV curve of the device, with the electrical details shown as an inset. The fabricated cell achieves an efficiency of 16.91% while maintaining good semitransparency,^46^ with an average visible transmittance of 18% across the 390–780 nm visible range. This corresponds to a light utilization efficiency (LUE) of 3%.

**Figure 6:**
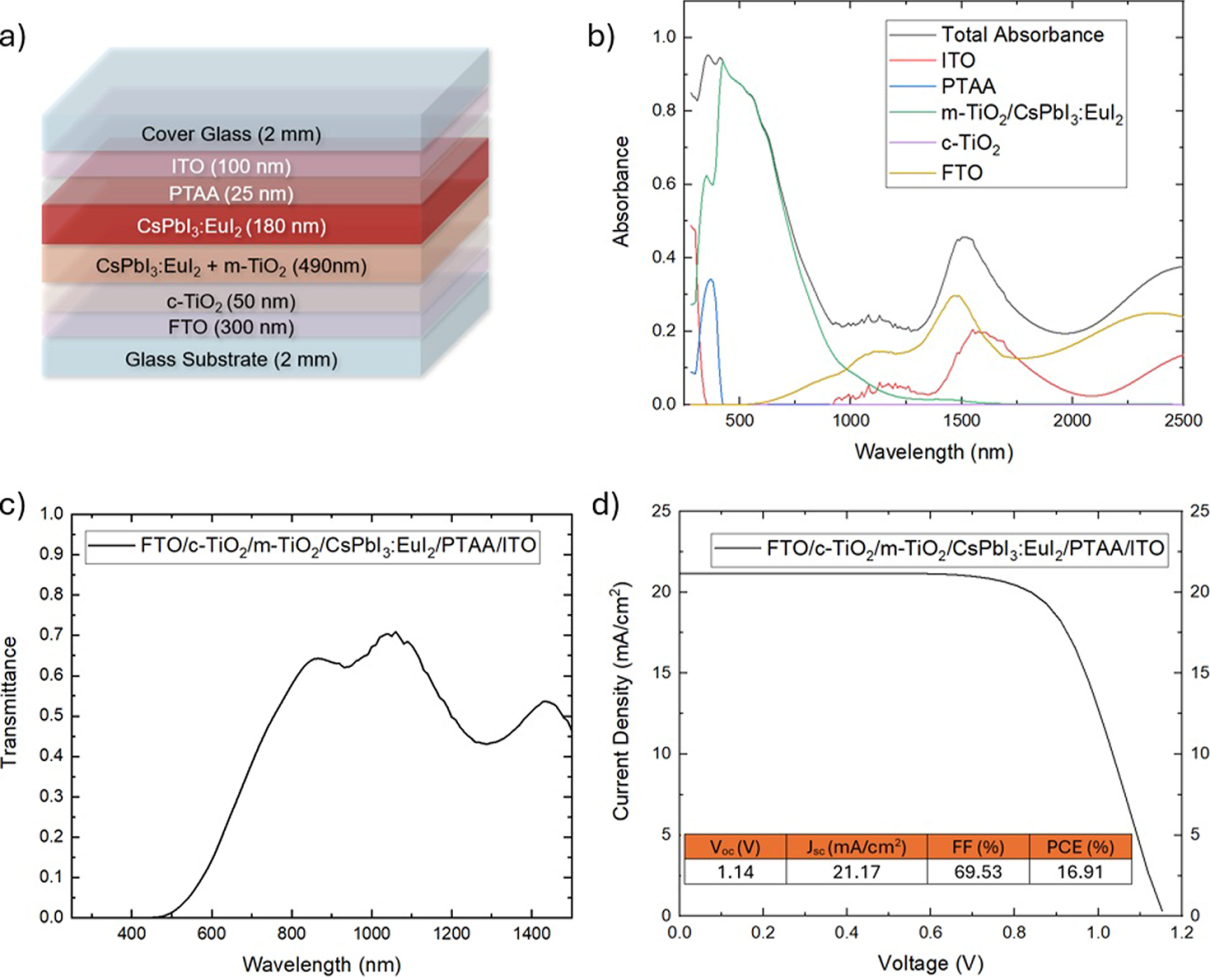
(a) Schematic of the simulated device. (b) Total absorbance and layer-resolved absorbance for single layer. The contribution by c-TiO_2_ is negligible at all wavelengths. (c) Transmittance of the entire device. (d) *J*–*V* curve of the device with the electrical parameters as inset.

In the model, the maximum power point tracking (MPPT) value is constant over time and equals 16.92 mV/cm^2^. Figure 7 shows the external quantum efficiency (EQE) values of the semitransparent device.

**Figure 7:**
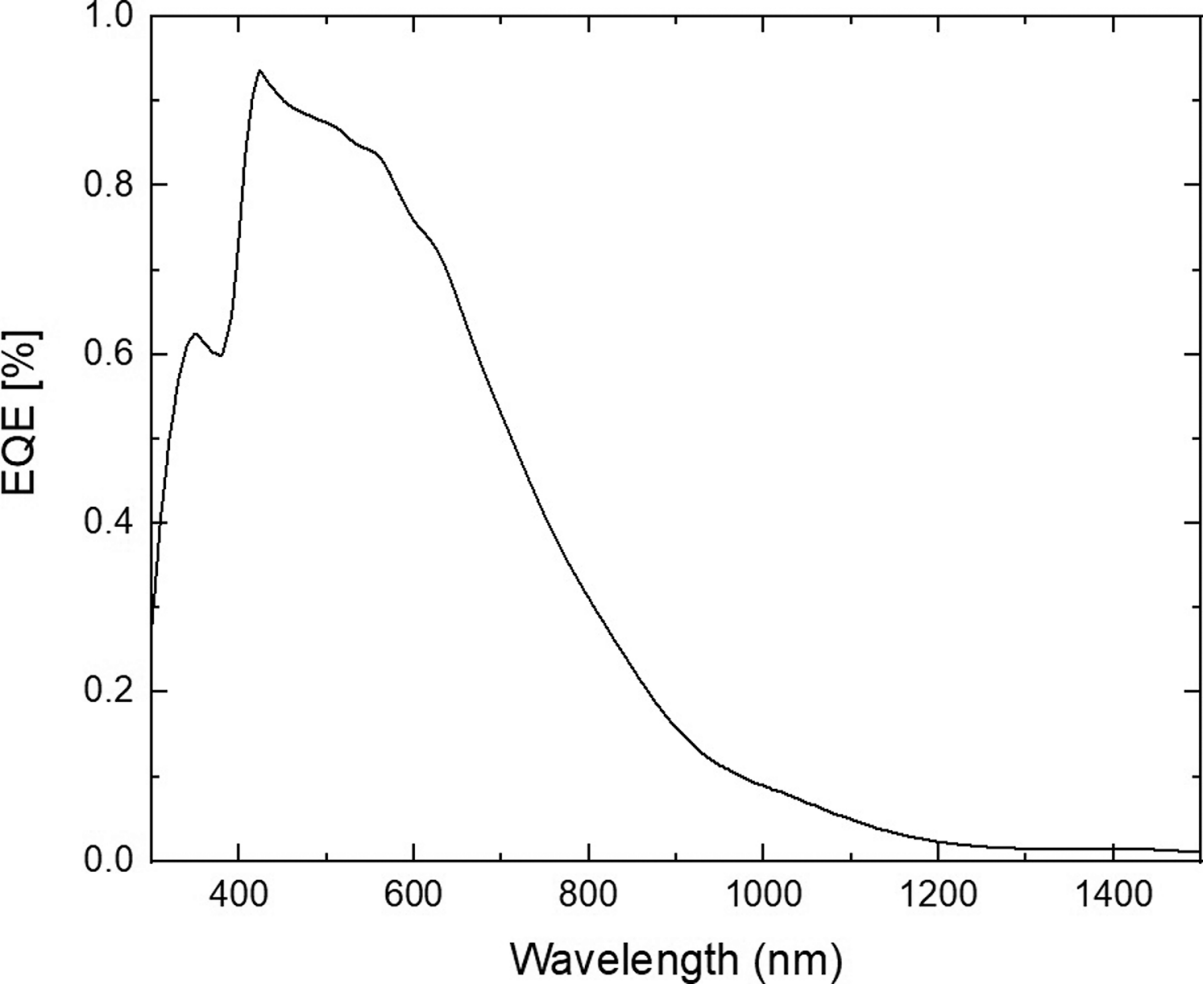
EQE data of the simulated semitransparent device.

A comparison with a device made with conventional TiO_2_ ETL is shown in Figure S3 of the supporting file.

## Conclusions

We have developed a process that successfully integrates CsPbI_3_:EuI_2_ perovskites into gig-lox TiO_2_, enabling their use as an active layer with built-in electron extraction functionality. The process yields a bilayer structure featuring a thin perovskite cap layer on the top and a perovskite-oxide blend beneath. This configuration preserves semitransparency while ensuring a loading of photoactive material for half of the overall volume. This structure exhibited reduced PL intensity compared to the reference CsPbI_3_:EuI_2_ deposited on glass, indicating efficient charge carrier injection into the porous TiO_2_ via a built-in potential established at the inner interfaces. Additionally, X-ray diffraction analyses revealed a gained adaptability of the perovskite material into gig-lox TiO_2_ as deduced by the strain relaxation found by measuring the lattice volume. Meanwhile, the confined pore space limits grain growth, which further helps to reduce lattice strain. Consequently , a shift of the bandgap to higher energies is observed, which is beneficial in applications requiring increased semitransparency. Finally, full device simulations based on the measured parameters prospect good device efficiency (better than using a conventional TiO_2_) while maintaining excellent semitransparency. These outcomes highlight the potential for applications in building integrated photovoltaics and agrivoltaics.

## Supplementary Material



## Notes

Thickness of samples at different speeds of spin coating; cross-FIB analysis highlighting the perovskite-infiltrated gig-lox TiO_2_ layer and the pure-perovskite capping layer; and transmittance and reflectance taken from the TiO_2_/FTO stack (PDF)
