# Supplementary material for: A Sputtered Gig-Lox TiO2 Sponge Integrated with CsPbI3:EuI2 for Semitransparent Perovskite Solar Cells
Source: J Phys Chem C Nanomater Interfaces. 2025 Aug 31;129(36):16338–46. doi: 10.1021/acs.jpcc.5c03520 (PMC12434809; doi:10.1021/acs.jpcc.5c03520)
Supplement: Supplementary file 1 [file jp5c03520_si_001.pdf]

A sputtered gig-lox TiO_2 sponge integrated with $CsPbI_3$:EuI_2 for semitransparent perovskite solar cells

C. Spampinato,[†,‡] G. Calogero,[*,†] G. Mannino,[*,†] S. Valastro,[†] E. Smecca,[†] V. Arena,[†] P. La Magna,[†] C. Bongiorno,[†] E. Fazio,[‡] and A. Alberti[†]

†National Research Council-Institute for Microelectronics and Microsystems (CNR-IMM), Zona Industriale - Strada VIII no. 5, Catania 95121, Italy
‡Dipartimento Scienze Matematiche e Informatiche, Scienze Fisiche e Scienze della Terra, Università Degli Studi di Messina, Messina, Italia

E-mail: gaetano.calogero@cnr.it; giovanni.mannino@cnr.it

Supporting information:

Thickness (nm)	Spin coating speed (rpm)
180	1000
178	2000
182	3000
181	4000
180	5000

Table S1. Thickness of samples at different speeds of spin coating.

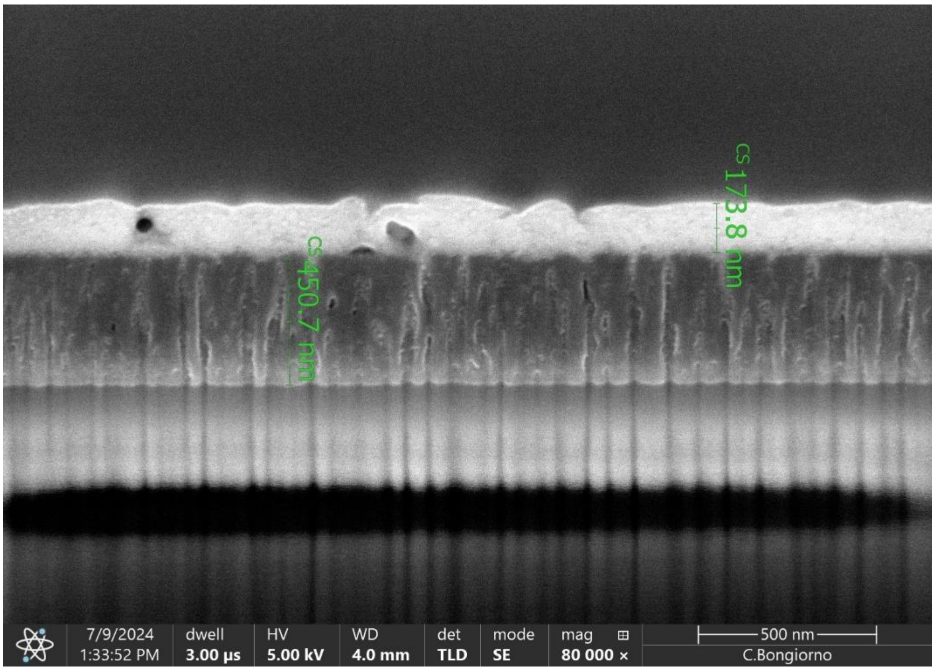

Figure S1. Cross-FIB analysis highlighting the perovskite-infiltrated gig-lox TiO2 layer (451nm) and the pure-perovskite capping layer (179nm).

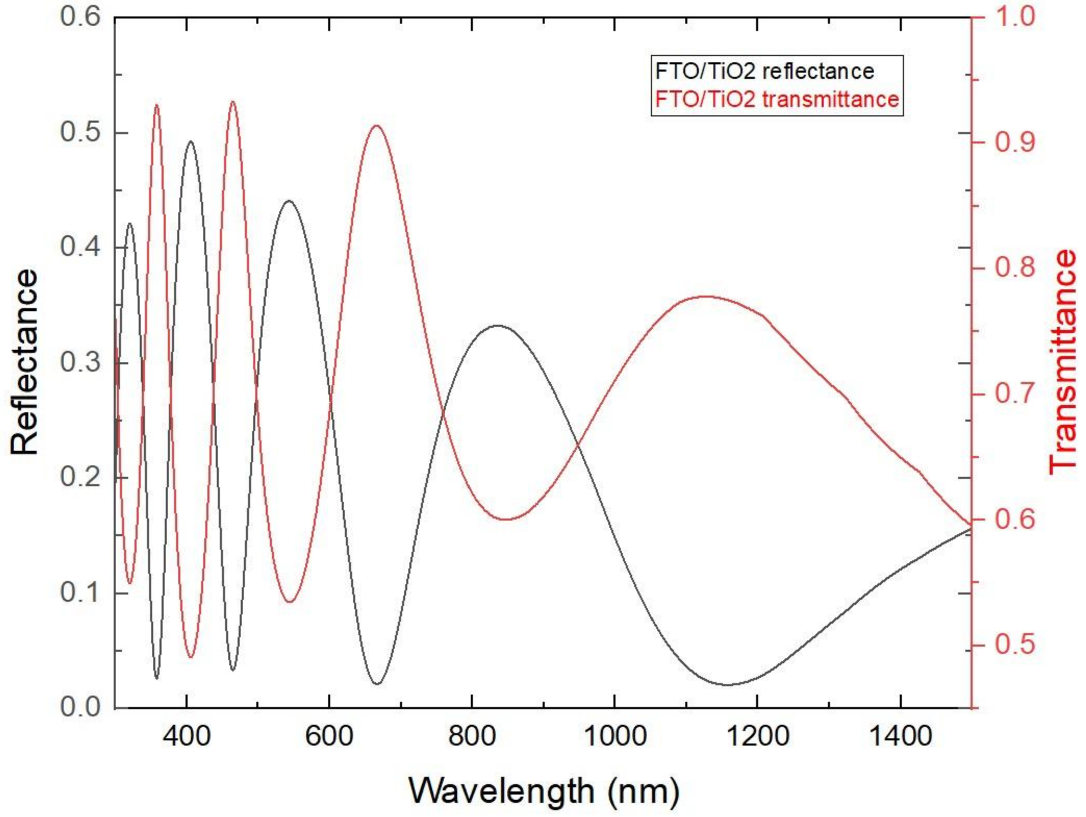

Figure S2. Transmittance and reflectance taken from the TiO_2/FTO stack.

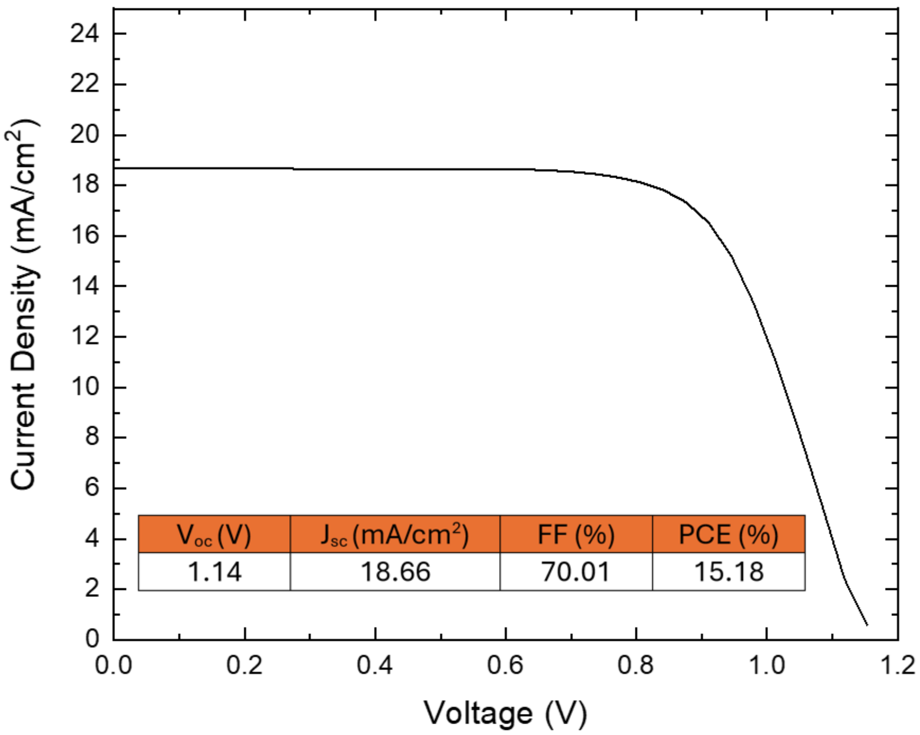

Figure S3. Simulated J-V curve of the conventional TiO2 device with the electrical parameters as reported in the inset.
